# Improving Cognition in Severe Mental Illness by Combining Cognitive Remediation and Transcranial Direct Current Stimulation: Study Protocol for a Pragmatic Randomized Sham-Controlled Multi-Center Trial (HEADDSET+)

**DOI:** 10.1192/j.eurpsy.2025.2167

**Published:** 2025-08-26

**Authors:** N. C. Buist, A. Poppe, B. Ćurčić-Blake, G. H. M. Pijnenborg, L. van der Meer

**Affiliations:** 1 Clinical and Developmental Neuropsychology, University of Groningen, Groningen; 2 Department of Rehabilitation, Lentis Psychiatric Institute, Zuidlaren; 3 Department of BSCS Neuroscience, University Medical Center Groningen, Groningen; 4 Department of Psychotic Disorders, GGZ Drenthe, Assen, Netherlands

## Abstract

**Introduction:**

Individuals with severe mental illness (SMI) frequently experience challenges in daily life, often attributable to cognitive impairments. Cognitive rehabilitation interventions can be implemented to enhance thinking abilities and improve functional outcomes. Transcranial direct current stimulation (tDCS) is a non-invasive brain stimulation technique that may promote neural plasticity and therefore, may enhance learning.

**Objectives:**

This trial aims to determine whether individuals with SMI who need supported housing can improve cognitive and daily functioning after following cognitive remediation (CR). Next, this trial evaluates whether CR combined with tDCS will enhance the effect of CR alone. Lastly, this trial will investigate the subjective experience of the CR intervention. We expect that participants will improve in goal attainment and cognitive and daily functioning.

**Methods:**

In this pragmatic, triple-blinded, randomized, sham-controlled multi-center trial, we will compare the experimental group (CR + active tDCS) with the control group (CR + sham tDCS). 126 participants with SMI will receive 16-20 weeks of twice-weekly CR (32-40 sessions of 30-45 minutes) combined with active (N = 63) or sham tDCS (N = 63), separated over five cohorts. We will recruit participants aged between 18 and 65 with SMI residing in supported living facilities. Functional, cognitive, and clinical outcome assessments will be performed at baseline, post-16-week waiting period, post-treatment, and 6-month post-treatment. Additionally, post-treatment participants will be asked to engage in an in-depth interview to evaluate their meta-cognitive skills and subjective experience of the treatment.

**Results:**

Preliminary results from the post-treatment effects, along with insights from in-depth interviews conducted in the first cohort (N = 15) as well as post-16-week waiting period effects for goal attainment (including the second cohort, N ≈ 40) will be presented.

**Conclusions:**

This randomized controlled trial will investigate the efficacy of CR and tDCS in enhancing recovery in people with SMI. If the intervention proves to be effective, it has the potential to be implemented into standard care for service users requiring long-term support.

**Disclosure of Interest:**

None Declared

